# Interim Results of Rigicon Penile Prosthesis Implantation in a Single Center: The Largest Series in Türkiye

**DOI:** 10.5152/tud.2025.24122

**Published:** 2025-01-03

**Authors:** Muhammet İhsan Karaman, Rasim Güzel, Duygu Kirkik, Orhan Koca

**Affiliations:** 1 Department of Urology, Medistate Kavacık Hospital, İstanbul, Türkiye; 2 Department of Medical Biology, University of Health Sciences Hamidiye Faculty of Medicine, İstanbul, Türkiye

**Keywords:** 3-piece inflatable, inflatable penile prosthesis, penile implant, safety, Rigicon Infla10^®^

## Abstract

**Objective:**

To analyze data from patient information forms (PIFs) submitted to the manufacturer of a new 3-piece inflatable penile prosthesis (IPP), the Rigicon Infla10^®^, to summarize interim outcomes of 250 implantations in a single center, which is the largest series in Türkiye.

**Methods:**

A retrospective review of PIFs from 250 patients implanted with the IPP between January 2021 and December 2023 was performed to assess patient characteristics, surgical data, device durability, patient satisfaction, and rates of reoperation for any reason.

**Results:**

The mean ± SD (range) follow-up was 21.2 ± 8.7 (6-36) months and the mean ± SD patient age was 56.2 ± 9.1 years. The average surgical time was 47 minutes. Of the total, 2.4% of devices required removal or revision. Mechanical failure of the device was reported in 3 patients (1.2%). One patient (0.4%) required revision for noise during pumping. Two patients (0.8%) requested device removal because of dissatisfaction. No infections were observed in this series. A total of 97.6% of the Rigicon Infla10^®^ devices were free from explant or revision. The satisfaction rate based on a single question asked to the patient by the surgeon 6 months after surgery was 98.4%.

**Conclusion:**

All Rigicon Infla10^®^ IPPs implanted in the center prior to January 2024 were included in the retrospective analysis of volunteered PIFs. Initial durability from reoperation was demonstrated to be superior compared to other contemporary devices. Additionally, high satisfaction with treatment outcomes was reported by 92.4% of patients.

**Main Points**

- **Largest Series in Türkiye:** The study presents interim findings from the largest series of Rigicon Infla10^®^ IPP implantations conducted at a single center in Türkiye, with 250 patients evaluated over a 3-year period.
- **High Device Reliability:** The Rigicon Infla10^®^ demonstrated exceptional durability, with only 2.4% of cases requiring revision or removal due to mechanical issues or dissatisfaction, and no infections reported.
  **Innovative Device Features:** The Infla10^®^ AX model offers dual expansion (circumferential and longitudinal), improving patient outcomes for conditions like Peyronie’s disease or penile shortening.
- **Patient Satisfaction:** An impressive 92.4% of patients expressed high satisfaction, showcasing the procedure’s efficacy and the prosthesis’s design.
- **Robust Methodology:** A retrospective analysis of standardized patient information forms (PIFs) ensured comprehensive data collection, minimizing bias and maximizing reliability.

## Introduction

Erectile dysfunction (ED) is the inability to achieve or maintain an erection sufficient for sexual activity, and this condition negatively affects the quality of life of patients by significantly impacting both physical and psychosocial health.^1,2^ Over the past 50 years, inflatable penile prostheses (IPP) have emerged as a highly successful treatment option, particularly for men with ED who do not respond to medical therapies.^3,4^ These prostheses have revolutionized the treatment landscape for ED, providing reliable solutions where medications fall short.

In the last 50 years, many types of prostheses have been developed.^4,5^ While all types provide penile rigidity, inflatable prostheses are favored for their ability to also allow penile flaccidity, resulting in a more natural appearance.^6^ Among the most established and widely used brands are the American Medical Systems (AMSs) 700 and the Coloplast Titan.^4,7^ However, the introduction of a new IPP by Rigicon Inc., 5 years ago, has added another strong contender to the market.^4^ Manufactured in the United States, Rigicon Infla10^®^ is now one of the most implanted prostheses globally and holds CE (Conformité Européenne) certification and is used in many countries, including Australia, the Middle East, Colombia, Ecuador, Argentina, Türkiye, as well as European countries such as Spain, Germany, the United Kingdom, France, and Italy. This new device to the range of available treatments offers patients more options and has contributed to advancements in the field of ED management.

This study aims to present interim findings from the largest series of Rigicon Infla10^®^ IPP implantations conducted at a single center in Türkiye. The evaluation specifically focuses on examining instances of removal or reoperation due to mechanical failure, medical complications, or infections related to Rigicon’s advanced penile prosthesis technology.

## Material and Methods

### Study Population

This study was reviewed and approved by the Medipol University under approval number 740. In this study, 250 patients who underwent IPP implantation at Private Kavacık Hospital between January 2021 and December 2023 were retrospectively analyzed. All data were collected through standardized patient information forms (PIFs) to ensure uniformity. To minimize selection bias, patients were consecutively included based on the order of their treatment during the study period. Additionally, data were obtained from patients who were reachable either by phone or through face-to-face interviews, ensuring thorough data collection and verification. All patients included in the study were selected from patients diagnosed with ED (impotence) who received an IPP for the first time and accepted the Rigicon Infla10^®^ 3-piece IPP as treatment. All surgeries were performed by 2 surgeons with over 20 years of professional experience, ensuring high surgical expertise. In this study, a strict non-touch technique was applied to all patients, minimizing the risk of contamination during surgery. Additionally, all prostheses were coated with gentamicin and rifampicin antibiotics preoperatively to provide robust antimicrobial coverage. Cefazolin was administered for Gram-positive bacteria, Ceftriaxone for gram-negative bacteria, and Fluconazole for antifungal coverage. Postoperatively, patients were prescribed amoxicillin/clavulanic acid and cefixime for 10 days to further reduce infection risks. During surgery, the patients were fitted with Rigicon’s Infla10^®^ X and Infla10^®^ AX IPP models according to their anatomical structure. The Infla10^®^ X uses cylinders that expand circumferentially when inflated, are 12, 15, 18, 20, 22, and 24 cm in length, and have an uninflated diameter of approximately 12 mm. The Infla10^®^ AX is similar but expands both circumferentially and longitudinally and is available in the same lengths. The appropriate model and length are selected according to the specific anatomical requirements of the patients. The pump and cylinders are pre-connected but not pre-filled. These models are available in infrapubic and penoscrotal versions and differ only in the length of the tube between the pump and the cylinders. Data were evaluated in detail for 3-year outcomes. Information from PIF forms completed at the time of each implantation was used as the data source. Patient privacy was maintained, and names were anonymized before analysis. Data included demographic characteristics (age, height, weight, cause of ED), and date of surgery. ^8^^,^^9^

### Patient Satisfaction

Patient assessment was performed through telephone or face-to-face interviews.^10^ A single question was posed to the patient by the surgeon 6 months after surgery, and responses were obtained using a 5-point Likert scale (1: dissatisfied; 2: somewhat dissatisfied; 3: neutral; 4: satisfied; 5: very satisfied). Satisfaction level was defined as patients scoring 4 or higher.^11^

### Statistical Analysis

Statistical analysis for this study was conducted using the SPSS 25.0 program (IBM SPSS Corp.; Armonk, NY, USA). Descriptive statistics were used to summarize demographic characteristics such as age, height, weight, and underlying causes of ED among the 250 patients. Surgical details including the date of operation, specific model and component sizes of the Rigicon IPP implants, surgical incision locations, and reasons for any revisions were also analyzed. Furthermore, Rigicon survival analysis was employed to assess the long-term outcomes over a 3-year period, while patient and partner satisfaction levels were evaluated through structured telephone or face-to-face interviews.

## Results

The study included 250 patients who underwent IPP implantation (Table 1). The mean age of the patients was 56.2 years with a SD of 9.1 years, ranging from 31 to 72 years. 

The follow-up duration averaged 21.2 months, with a SD of 8.7 months, and ranged from 6 to 36 months. This provides a substantial period for assessing the outcomes and potential complications post surgery.

The average operation duration was 47 (35-72) minutes. A vast majority (96%) of the surgeries utilized the penoscrotal incision location, with only a small fraction (4%) using the infrapubic approach. This decision was made due to patient-specific anatomical considerations or patient’s and/or surgeon’s personal preference.

The underlying causes of ED among the 250 patients who underwent IPP implantation at Kavacık Private Hospital are detailed in Table 2. The most common cause of ED in this cohort was diabetes mellitus, accounting for 40.4% of cases. Vascular insufficiency was the second most prevalent cause, affecting 21.2% of patients, followed by radical prostatectomy at 18.8%. Other notable causes included hypertension (4.8%), Peyronie’s disease (4.4%), and priapism (2%). Additionally, 8.4% of the cases were attributed to unspecified causes and psychogenic etiology. This distribution highlights the diverse etiologies of ED and underscores the importance of tailored treatment approaches for patients undergoing IPP implantation. 

The corporal measurements, including the lengths of the rear tip extenders used during the operation, are detailed in Table 3. The total corporal length averages 18.2 cm with a SD of ±2.7 cm for both the left and right sides. Additionally, the rear tip extenders used during the procedures measured approximately 2.4 cm. These detailed measurements are crucial for accurately assessing the anatomical requirements of patients and for planning the implantation of the penile prosthesis.

**Figure 1.**
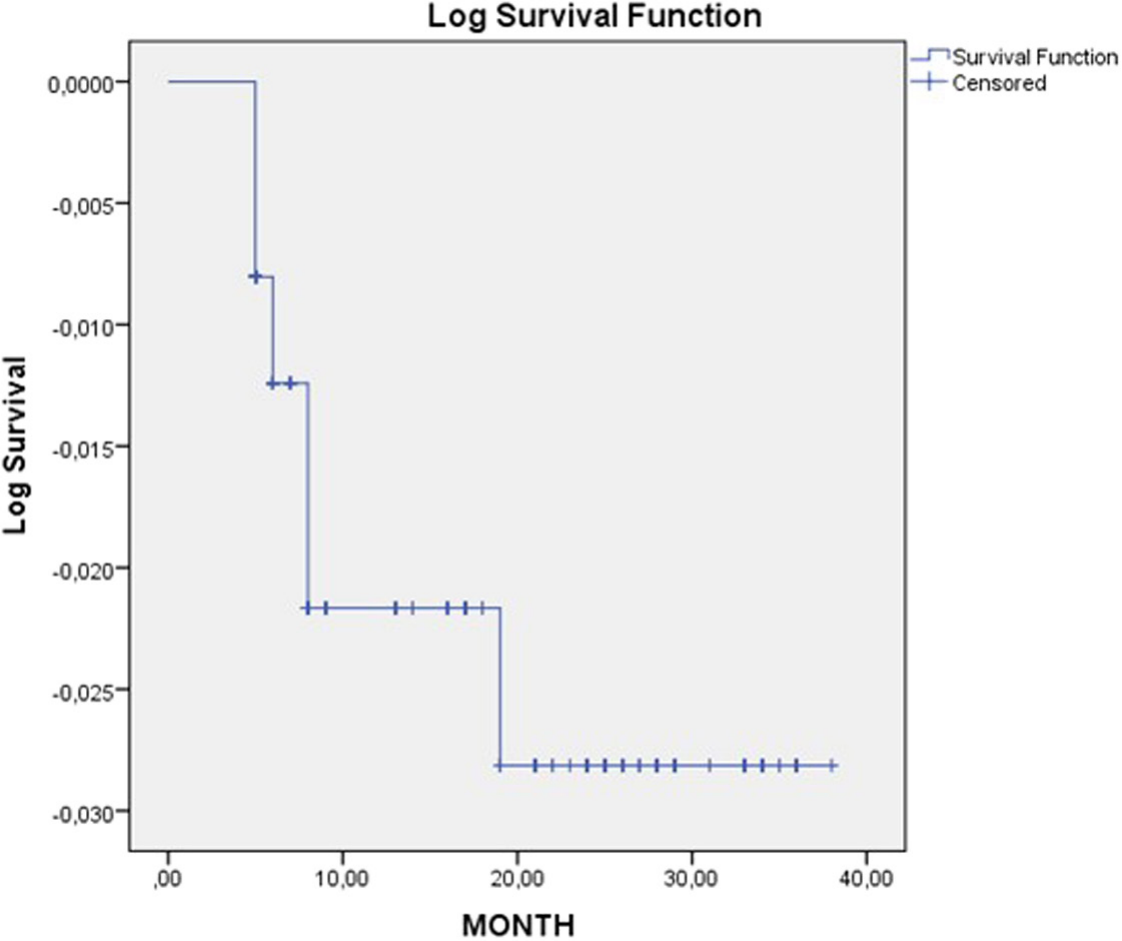
Kaplan–Meier calculations of survival from removal or revision were 97.6% at 36 months.

**Figure 2.**
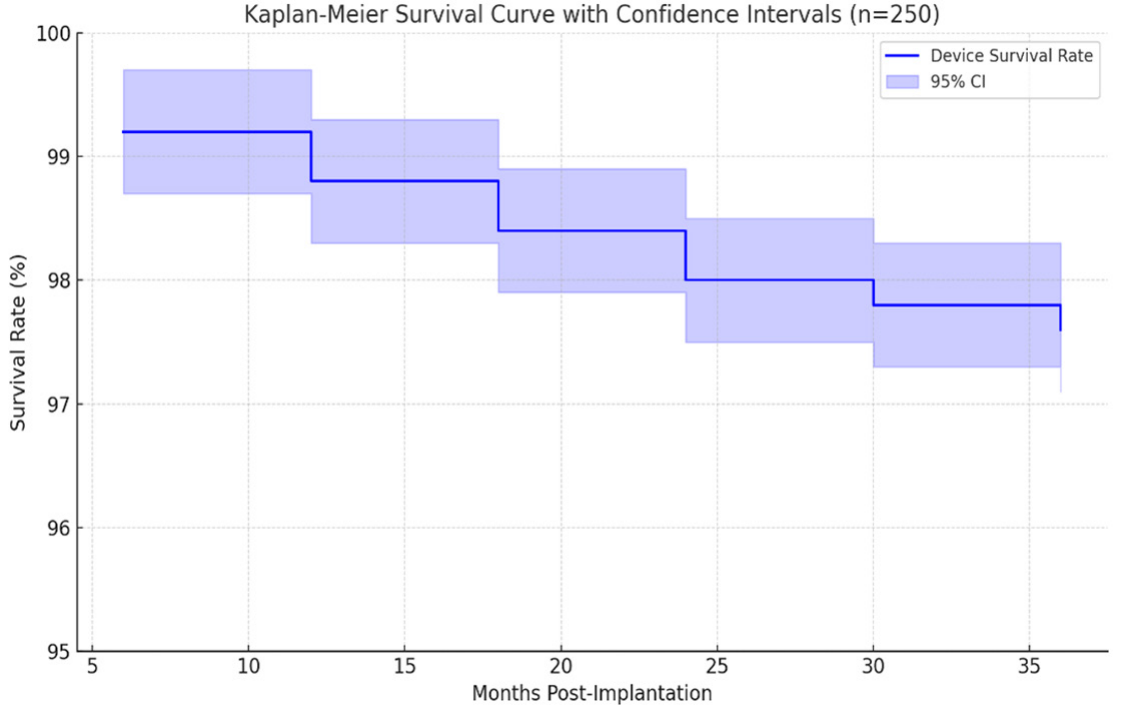
Kaplan–Meier survival curve of penile prosthesis device durability over 36 months with 95% CI.

Out of the 250 patients, 4 required revision and 2 required removal of their prosthesis, making a total of 6 cases (2.4%). The most common reason for revision/removal was mechanical failure, accounting for 1.2% of the cases, including issues such as fluid loss. Another factor related to revision/removal was patient dissatisfaction, affecting 0.8% of the patients, which could involve subjective factors related to the patient’s expectations or experiences post surgery. Noise from the pump was reported in 0.4% of the patients, indicating that while rare, mechanical issues can arise that impact patient comfort or device performance. This suggests that a small percentage of patients experience complications that necessitate further surgical intervention. The results of patient satisfaction and outcomes are summarized in Table 4. In Figure 1, the Rigicon curve represents the proportion of patients who maintained their IPP without any surgical intervention requiring removal or revision over a period of 36 months. The survival rate is shown as a percentage on the y-axis, and time is shown in months on the x-axis. The key finding here is that 36 months (3 years) after implantation, 97.6% of patients had not experienced any event requiring removal or revision of their prosthesis. This high survival rate indicates that the Rigicon Infla10^®^ IPP is durable and reliable, with only a small percentage (2.4%) of patients reporting problems requiring surgical intervention during the 3-year follow-up period. The Kaplan–Meier survival curve (Figure 2) provides a visual representation of the device’s survival rate over a 36-month follow-up period, accompanied by a 95% CI. The blue line represents the percentage of patients who retained their penile prosthesis without requiring revision or removal. The shaded blue area around the line indicates the 95% CIs, offering an estimate of the variability in survival rates.

**Figure 3.**
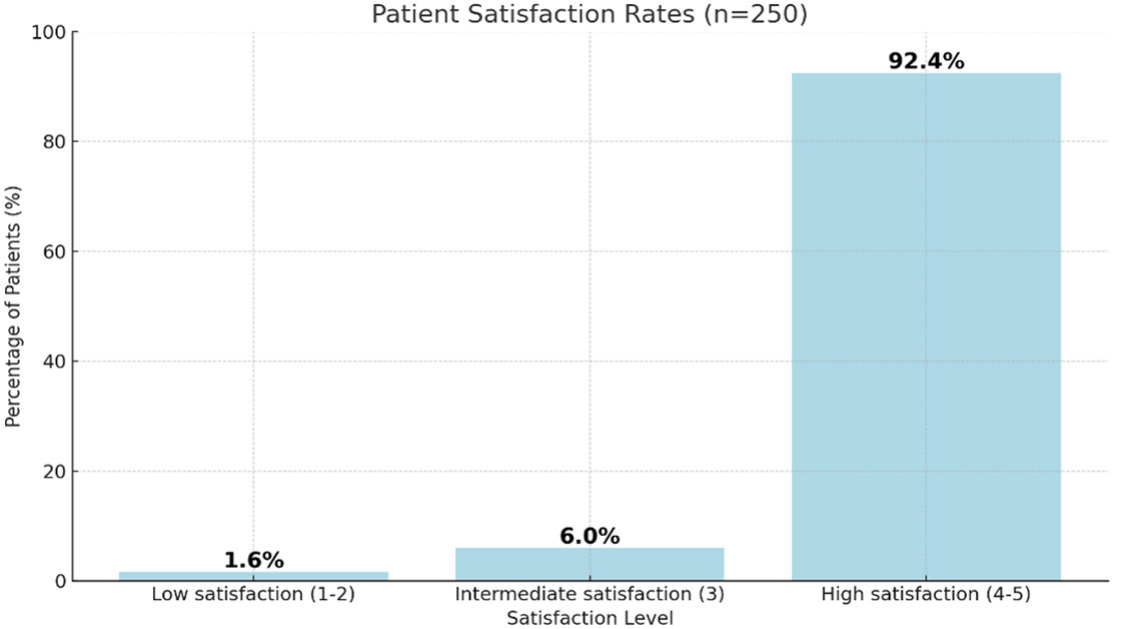
Satisfaction and outcomes of the patients.

Out of 250 patients assessed, 4 patients (1.6%) reported low satisfaction, scoring between 1 and 2 on the Likert scale. Intermediate satisfaction, with a score of 3, was reported by 15 patients, accounting for 6% of the total. The majority of the patients, 231 individuals (92.4%), expressed high satisfaction, with scores ranging from 4 to 5. This indicates that the vast majority of patients were satisfied with the outcomes of their treatment (Figure 3).

## Discussion

Penile prosthesis surgery is frequently performed for patients with ED who do not respond to medical treatments.^12^ The surgical suitability of the prosthesis, its ability to mimic physiological erection, and ease of use are crucial factors contributing to the satisfaction of both patients and their partners. Although penile prosthesis implantation is the oldest modern option for treating ED, it remains a vital component of ED treatment.^13^ Advances in prosthesis design and improvements in implantation techniques have significantly increased patient satisfaction and extended the lifespan of prostheses. Penile prostheses achieve high satisfaction rates by providing a rapid, rigid, and reliable erection.^5,14^ It offers numerous advantages, including high success rates, long-term mechanical reliability, and the ability to achieve success without the need for injections or pills. Additionally, penile prostheses are particularly effective in patients with Peyronie’s disease or penile fibrosis, making them a highly successful treatment options.^15-19^

The interim findings from our study on the Rigicon Infla10^®^ IPP implantations at a single center in Türkiye provide critical insights into the efficacy, patient satisfaction, and complications associated with this new generation of IPP. Our study evaluated 250 patients, highlighting the demographic characteristics, surgical details, patient satisfaction, and reasons for prosthesis revision or removal, offering a comprehensive understanding of the performance of the Rigicon Infla10^®^ IPP over a 3-year follow-up period.

In this study, the demographic analysis showed a mean patient age of 56.2 years, reflecting the typical age range of men seeking treatment for ED. The primary cause of ED in our cohort was organic in nature, accounting for more than 90% of cases. This high prevalence underscores the necessity for effective interventions like IPPs, particularly for patients with physiological and anatomical impairments that do not respond to medical therapies. 

The low rate of revision or removal (2.4%) among our patient population is particularly noteworthy. Mechanical failure (1.2%), patient dissatisfaction (0.8%), and noise from the pump (0.4%) were the reasons for revision/removal. The mechanical failure rate of 1.2% aligns with the lower end of the spectrum reported in the literature, indicating the robustness of the Rigicon Infla10^®^ device. The primary cause of dissatisfaction was subjective, suggesting that patient education and setting realistic expectations are crucial components of preoperative counseling. The survival curve shows that 97.6% of patients retained their implant without needing surgical intervention for removal or revision, indicating a high success rate of the procedure. Only a small fraction, 2.4% of patients, encountered issues that required surgical attention within 3 years post implantation. This data suggest that the Rigicon Infla10^®^ IPP is an effective and dependable solution for patients, ensuring long-term satisfaction and minimal complications. The Kaplan–Meier survival curve demonstrates the device’s durability for penile prosthesis implantation over a 36-month follow-up period. As shown in the figure, 97.6% of the devices remained functional without requiring revision or removal at the end of 36 months. The CIs provide additional context, indicating the reliability of these survival estimates. The low rate of revisions and removals, as shown in the survival curve, supports the overall durability and effectiveness of the prosthesis over time. This finding is consistent with the mechanical reliability observed in other contemporary penile prostheses.

When comparing our findings with other well-established IPPs like AMS 700 and Coloplast Titan, the Rigicon Infla10^®^ appears to be a strong competitor. Previous studies on AMS 700 and Coloplast Titan have reported revision rates ranging from 2% to 5%, primarily due to mechanical failures or infections.^4,20,21^ Studies like those conducted by Atri et al^7^ and other reviews have shown no significant difference in prosthesis durability or survival between the 2 devices, though Coloplast Titan exhibited slightly increased axial rigidity in some cases. Our study’s low revision rate suggests that the Rigicon Infla10^®^ may offer superior reliability or that it is on par with existing models, which is promising for future clinical practice. Additionally, our study is notable for the absence of infections, a common complication associated with IPP implants. Several factors could contribute to this positive outcome, including improved surgical techniques, rigorous sterilization protocols, and effective postoperative care in a single-center setting. This lack of infections enhances the overall appeal of the Rigicon Infla10^®^, indicating that it might provide a safer alternative with fewer complications.

The low percentage of patients reporting low satisfaction (1.6%) or moderate satisfaction (6%) emphasizes the overall success of the procedures performed. In this study, a single-question Likert scale was preferred because of its suitability for clinical practice and ease of use. However, the contribution of more comprehensive assessment tools is recognized. As our study was focused on interim analysis, the structure of this simple method allowed us to quickly and effectively assess patient satisfaction during follow-up. In future studies, we plan to use more detailed assessment methods such as the International Index of Erectile Function in order to analyze patient outcomes in more depth and to extend the scope of the findings. Our results may indicate that the surgical techniques used, postoperative care, and patient education provided during the recovery process were good. However, the small percentage of patients reporting lower satisfaction should not be ignored. It is important to understand the factors contributing to their dissatisfaction. Reasons for this dissatisfaction may include the fact that patients may not see glans enlargement after surgery as they did with the previous natural erection, that they will feel a thrill in their hand during pumping, and that they will feel the presence of the implant in the penis by hand when the implant is deflated. Emphasizing such information in the pre-op visit may increase the postoperative satisfaction rate. Clearly communicating such details to patients preoperatively, managing expectations, and creating awareness about the situations they will encounter in the postoperative period can play a critical role in increasing patient satisfaction. In conclusion, while the results are highly positive overall, continuous evaluation and improvement are necessary to address the minority of cases with lower satisfaction and to sustain the high standards demonstrated in this study. 

The introduction of Rigicon Infla10^®^ has provided patients with more options, potentially driving advancements in clinical outcomes and satisfaction. Our data suggest that the Rigicon Infla10^®^’s design and functionality meet patient and clinical needs effectively, contributing to its rapid adoption globally. The Rigicon Infla10^®^ prosthesis introduces several innovative design features that differentiate it from other penile prostheses on the market. One key advancement is the dual expansion capability of the Infla10^®^ AX model, which allows for both circumferential and longitudinal expansion. This dual expansion not only enhances the girth and length of the prosthesis during inflation, mimicking a more natural erection, but also provides significant benefits for patients with conditions such as Peyronie’s disease or post-surgical penile shortening. Additionally, the pre-connected yet not pre-filled components of the device offer greater flexibility during implantation, allowing surgeons to make adjustments based on patient-specific anatomy. Combined with its high mechanical reliability and durability, evidenced by a low 2.4% revision/removal rate over 36 months, the Rigicon Infla10^®^ offers a cutting-edge solution that competes strongly with established models like the AMS 700 and Coloplast Titan. These features contribute to both high patient satisfaction and improved clinical outcomes, solidifying its role in modern ED management.

The safety and effectiveness of the IPP, Rigicon Infla10^®^, were assessed in our study using data from PIFs. The findings revealed a low risk of prosthesis, with only 2.4% of patients needing removal or revision at the 21.2-month follow-up. Comparatively, Rigicon Infla10^®^ appears to be equal to or even superior to other well-established IPPs such as AMS 700 and Coloplast Titan in terms of mechanical reliability and patient satisfaction. The findings suggest that Rigicon Infla10^®^ offers a promising alternative for patients with ED and provides effective solutions where medications have failed. In conclusion, Rigicon Infla10^®^ IPP has demonstrated excellent performance and high patient satisfaction, making it a strong contender in the field of ED management. The results of this study support the continued use and adoption of Rigicon Infla10^®^ and emphasize the importance of ongoing evaluation to maintain and improve patient outcomes.

## Figures and Tables

**Table 1.** Demographic Characteristics and Summary Statistics of Patients Undergoing Rigicon IPP Implantation

	**Mean ± SD**	**Min-Max**
Age, years	56.2 ± 9.1	31-72
Follow-up, months	21.2 ± 8.7	6-36
**Number of patients**		250
**Operation duration**		47 min
	**Number of patients**	**Percentage (%)**
Surgical incision location		
Penoscrotal	240	96
Infrapubic	10	4
Model of implanted Rigicon Infla10^®^ **IPP**		
Infla10^®^ AX	146	58.4
Infla10^®^ X	104	41.6

**Table 2.** Causes of ED of Primary Patients, n, (%)

**Etiology of ED**	**n**	%
Hypertension	12	4.8
Priapism	5	2
Peyronie’s disease	11	4.4
Diabetes mellitus	101	40.4
Vascular insufficiency	53	21.2
Radical prostatectomy	47	18.8
Other	21	8.4

**Table 3.** Corporal Measurements

**Corporal Measurement**	***L *(mean ± SD)**	***R* (mean ± SD)**
Total, cm	18.2 (±2.7)	18.2 (±2.7)
Rear tip extenders, cm	2.4 cm	2.4 cm

**Table 4.** Reason for Prosthesis Revision/Removal—2.4%

Mechanical failure (including 2 patients with fluid loss and 1 patient with spontaneous erection of the device)	3	1.2%
Patient dissatisfaction	2	0.8%
Noise from the pump	1	0.4%
Total	6	2.4%
